# The Emerging Use of *In Vivo* Optical Imaging in the Study of Neurodegenerative Diseases

**DOI:** 10.1155/2014/401306

**Published:** 2014-07-23

**Authors:** Aileen P. Patterson, Stephanie A. Booth, Reuben Saba

**Affiliations:** ^1^Molecular PathoBiology Unit, Public Health Agency of Canada, National Microbiology Laboratory, 1015 Arlington Street, Winnipeg, MB, Canada R3E 3R2; ^2^Department of Medical Microbiology and Infectious Diseases, Faculty of Medicine, University of Manitoba, 730 William Avenue, Winnipeg, MB, Canada R3E 0W3

## Abstract

The detection and subsequent quantification of photons emitted from living tissues, using highly sensitive charged-couple device (CCD) cameras, have enabled investigators to noninvasively examine the intricate dynamics of molecular reactions in wide assortment of experimental animals under basal and pathophysiological conditions. Nevertheless, extrapolation of this *in vivo* optical imaging technology to the study of the mammalian brain and related neurodegenerative conditions is still in its infancy. In this review, we introduce the reader to the emerging use of *in vivo* optical imaging in the study of neurodegenerative diseases. We highlight the current instrumentation that is available and reporter molecules (fluorescent and bioluminescent) that are commonly used. Moreover, we examine how *in vivo* optical imaging using transgenic reporter mice has provided new insights into Alzheimer's disease, amyotrophic lateral sclerosis (ALS), Prion disease, and neuronal damage arising from excitotoxicity and inflammation. Furthermore, we also touch upon studies that have utilized these technologies for the development of therapeutic strategies for neurodegenerative conditions that afflict humans.

## 1. Introduction

The ability to image cells, tissues, and whole animals has been at the forefront of medical technological advance since the advent of the first microscope and has resulted in the evolution of various imaging modalities, including X-ray, magnetic resonance imaging (MRI), ultrasound (US), positron emission tomography (PET), computed tomography (CT), and optical imaging. Optical imaging, in particular, employs light in the visible and near-infrared spectrum to visualize various cellular processes and has evolved from observing anatomical differences between tissue slices from a single time point to imaging multiple biological features longitudinally in a noninvasive manner in the same animal [1]. Additionally, the use of visible light photons for imaging is an attractive option as it is less harmful than repeated use of ionizing radiation utilized in most other medical imaging modalities.

Noninvasive or* in vivo* optical imaging is particularly advantageous for the study of neurodegenerative diseases. In contrast to conventional techniques that show an absolute reliance on access to brain tissue, which for the most part is only available postmortem,* in vivo* optical imaging permits the study of the tissue within the contextual influences of the intact animal. Moreover,* in vivo* optical imaging contributes towards the reduction in the number of animals used in basic research and drug development. For instance, the same animal can be imaged multiple times in order to monitor visually, often in real time, the progression or regression of infection or disease. In effect, an animal used in an experiment serves as its own control. This, in turn, avoids the need to sequentially sacrifice animals at different time points, allowing significant reductions in the number of animals used per study. With* in vivo* methods, fewer animals can deliver data with greater statistical significance. Additionally, more accurate animal models can be created that can bear the characteristics of a longitudinal study design, internal experimental control, and quantitative data. In short,* in vivo* optical imaging methods not only guide appropriate endpoint tissue sampling for histology or biochemical analysis but also benefit scientific inquiry and obey the principles of humane experimental techniques in medicine.

To date, most of the work that has been performed so far has utilized rodent models, most likely due to the availability of transgenic mice and the extensive knowledge of mice genetics and biology that exists. Therefore, in this review, we discuss how the emerging use of* in vivo* optical imaging in combination with reporter gene technology, particularly in mouse models, is contributing towards a better understanding of the intricate molecular underpinnings of neurodegenerative diseases and also how this technology is leading to the development of potential therapeutic options.

## 2. *In Vivo* Optical Imaging Capabilities

Several instruments are currently available to perform* in vivo* optical imaging, each with varying capabilities. Fluorescent and bioluminescent reporters are most commonly used, and most instruments are able to read data from both, including the NightOwl (Berthold Technologies),* In Vivo* imaging systems (Bruker), iBox Scientia Small Animal Imaging System (UVP), and the PhotonIMAGER (Biospace Lab). As well, the Mousepod is an accessory for the Odyssey CLx Infrared Imaging System (Li-Cor Biosciences). Several optical imaging systems are also able to be used in conjunction with other medical imaging modalities (MRI, PET, and CT) such as the IVIS series and FMT series of imaging systems (Perkin Elmer). In fact, systems are now being produced with integrated medical imaging, such as the IVIS Spectrum CT (Perkin Elmer), which has a built-in microCT. While these systems provide invaluable information, the mode or method of sedation of experimental animals used can exclude some research studies, such as those involving the sleep-wake cycle and the examination of the physiology of the immune system [2]. Recently, optical imaging of nonsedated animals by way of the In Actio Module for the PhotonIMAGER (Biospace Lab) through rapid acquisition of photons has been developed as a means of addressing this limitation. When choosing an instrument for* in vivo* optical imaging, it is important to consider the method of light detection and the software used to analyze images. Due to their high sensitivity, cooled CCD cameras are most often used. In fact, all of the above-mentioned instruments employ the use of a CCD camera except the Odyssey CLx Infrared Imaging System, which uses the nearly equivalent avalanche photodiode. As well, the software capabilities should be considered depending on the experiment. When two or more reporters are used with different emission wavelengths or tissue autofluorescence is an issue, spectral unmixing can be used to tease apart the different wavelengths. Imaging of several animals simultaneously can be performed on instruments that come equipped with a multiple mouse manifold to deliver anesthetic gas, such as the IVIS series from Perkin Elmer, which decreases the technician hands on time required. Alternatively, instruments without a multiple manifold can still be used to image several mice under injectable anesthetic, providing they fit in the CCD field of view; however, signal can only be measured for each mouse if the software is able to define multiple regions of interest (ROIs) for photon measurement. Multiple ROI capabilities are also of importance when the reporter used differentially localizes to multiple regions of the animal or multiple probes are used.

## 3. Optical Imaging Reporters

**Figure 1:**
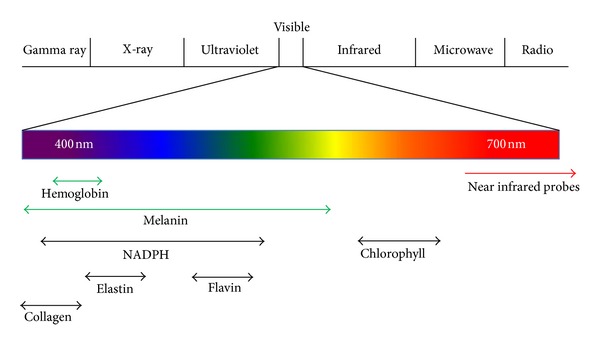
General location of absorption, tissue autofluorescence, and near infrared probes on the visible light spectrum. Factors contributing to tissue absorption (green arrows) and autofluorescence (black arrows) are indicated below.

**Figure 2:**
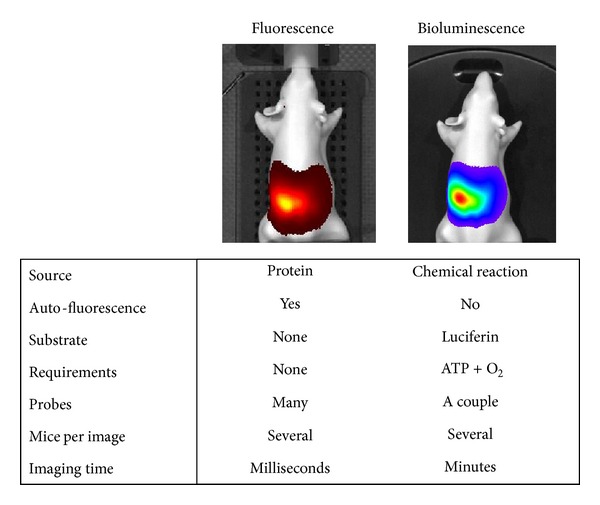
Comparison between fluorescent and bioluminescent reporters for use in* in vivo *optical imaging. The luciferase used for comparison in this figure is firefly luciferase.

Reporting the location and expression of molecular signals for optical imaging requires reporters that emit light; two of the most commonly used are fluorescent and bioluminescent reporters. Fluorescence relies on a variety of excitation and emission wavelength filter pairs for varying fluorescent reporters, whereas bioluminescence requires a substrate to complete the biochemical reaction to produce light [3]. Both methods of light generation possess inherent advantages and disadvantages during experimental setup, and, moreover, data analysis and reporter choice must be determined based on the requirements of the experiment(s) to be performed (Figure 1). A general limitation of visualizing fluorescent light in optical imaging is endogenous light absorption, which can be easily illustrated by holding different colour laser pointers to one's fingers and examining the degree of light transmission through the tissue. Green light results in little to no light transmission through tissue, whereas red light is more easily transmitted. This is due to endogenous absorption of light by hemoglobin and melanin in the lower part of the visible spectrum limiting the depth of light penetration (Figure 2) [4]. Therefore, animal positioning during imaging is of the utmost importance and must be adjusted so that the light signal is placed closest to the camera detector. In addition, multimodal imaging that combines photon information with structural information generated by MRI or CT, for example, plus the application of algorithms is providing improved ways to enhance spatial resolution and to reconstruct 3D models of light production within tissues.

## 4. Fluorescent Reporters

Fluorescent proteins absorb light photons at a wavelength specific to the protein, which then excites electrons to a higher energy state. As the electrons return to ground state, energy is released as light at a different wavelength generating a colour on the visible spectrum [5]. Imaging with fluorescent protein reporters has several advantages. Firstly, experimental setup is relatively easy, as once a reporter with certain fluorescence is chosen, it is integrated into the animal and imaged with the corresponding excitation/emission wavelengths for that fluorophore. Secondly, there are many fluorescent reporters available that emit light at varying wavelengths throughout the visible and near infrared spectrum. This enables multiple reporters to be used simultaneously by choosing fluorescent proteins with little spectral overlap. Although the setup is relatively easy, it can be difficult to interpret fluorescent data. Autofluorescence of skin, fur, and tissue, due to several cellular components, including NADPH, flavin coenzymes, elastin, and collagen, can interfere significantly with signal from fluorescent reporters if emission wavelengths overlap (Figure 2) [6]. Additionally, chlorophyll present in standard mouse food autofluoresces thus interfering with many common reporters [7]. To compensate for autofluorescence, software has been developed with advanced mathematical modeling to separate the sources of different wavelengths. This particular feature is referred to as spectral unmixing; nevertheless, many optical imaging instruments and software lack this capability [8, 9]. While fluorescent proteins have been traditionally used, nonprotein based fluorophores commonly used in cellular imaging, such as fluorescein and CyDyes, are alternative fluorescent probes for use in* in vivo* optical imaging, and recently, quantum dots have been developed for optical imaging. Quantum dots are small, inorganic nanoparticles that emit a specific wavelength of light depending on their size, from ultraviolet to near-infrared, and can be conjugated to molecules that localize fluorescence to an area of interest [10]. They offer increased brightness and stability over fluorescent proteins and providing a means to manipulate the wavelength emitted by simply altering the size of the nanoparticle. However, since nonprotein based reporters cannot replicate* in vivo*, they cannot be made into fusion proteins to monitor promoter activity.

## 5. Bioluminescent Reporters

Bioluminescence is most commonly used for* in vivo* optical imaging and refers to the light that is generated by a chemical reaction between the substrate, luciferin, and oxygen, in which luciferase acts as the enzyme to accelerate the reaction [11]. When the electron of this reaction product returns to ground state, energy is emitted in the form of light, similar to fluorescence. There are several different bioluminescent reporter systems, each isolated form a different species and generating light at varying wavelengths (summarized in Table 1). Whereas fluorescence data analysis can be difficult to interpret due to tissue autofluorescence, there is no endogenous tissue bioluminescence; therefore, all detected light directly results from the luminescent reporter. Nevertheless, the experimental setup is slightly more challenging compared to fluorescence. As a luciferin substrate is required for most of the bioluminescent chemical reactions, and is not endogenous to animal models, it must be supplied exogenously. Therefore, experimental consistency is important to produce comparable results. To establish the optimal dosage of luciferin and the optimal time to image the animal after injection of the substrate, a kinetic curve is initially generated. While the need for a kinetic curve is only required once, it can become challenging when studying an experimental animal from birth to adulthood due to alterations in metabolism that can alter the kinetics of luciferin processing. Nevertheless, the fact that luciferin is able to cross the blood brain barrier (BBB) is especially pertinent for neuroimaging [12]. A recent advancement that may eliminate the use of exogenous luciferin in some models is the use of the bacterial luciferase (*lux*) gene cassette that contains both the luciferase and luciferin genes [13]. Light is generated in the location of the reporter without relying on the bioavailability and/or kinetics of the luciferin substrate. Besides the obvious increase in gene size required for engineering the cassette, there are several drawbacks that accompany this technique, including the lower degree of gene expression than traditional firefly luciferase and also the shorter emission wavelength (490 nm and 560 nm, resp.). The latter drawback may be a problem for deep tissue imaging due to the absorption of the signal [13]. In addition, the use of a secreted luciferase may increase the resulting signal, as the substrate no longer requires entry into the cell expressing the luciferase; however, this may also lead to diffusion throughout the body, thus preventing accurate localization of the signal.

## 6. *In Vivo* Optical Imaging in the Study of Neurodegeneration

The use of* in vivo *optical imaging technology is emerging as an important addition to the array of tools currently available for the study of neurodegenerative conditions. These diseases and disorders of the central nervous system (CNS) are characterized by the progressive loss of neuronal function and structure that eventually culminates in cell death. There are several different types of neurodegenerative diseases classified largely by the identity of the neuronal cell population that is afflicted. These include Alzheimer's disease, Parkinson's disease, Huntington's disease, amyotrophic lateral sclerosis (ALS), and Prion diseases. Their complex etiology is common amongst the majority of neurodegenerative diseases; they are not monogenic or polygenic diseases and pathogenesis is multifaceted by events that are, most often, independent of genetic mutations. At the molecular level, the events responsible for neurodegeneration include oxidative stress, axonal transport deficits, protein oligomerization and aggregation, calcium deregulation, mitochondrial dysfunction, neuron-glial interactions, neuroinflammation, DNA damage, and aberrant RNA-processing. One of the greatest risk factors for neurodegeneration is advanced chronological age, in combination with mitochondrial DNA mutation and oxidative stress damage. Due to the extended life expectancy in the developed world, the prevalence of many of these diseases is expected to rise. Therefore, identification of tools that can assist in the rapid detection and quantitative assessment of the neuropathological status of diseased individuals is of the utmost importance, not only for diagnostic purposes but also for the development and evaluation of effective therapeutic options.

**Figure 3:**
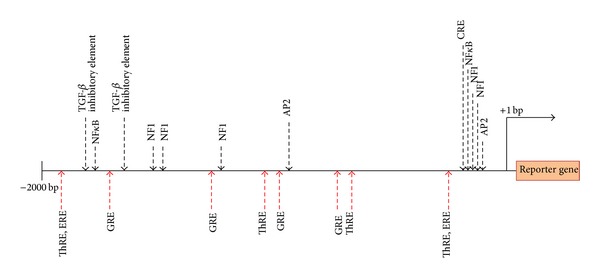
Putative response element and transcription factor binding sites within the mouse GFAP 5′-upstream region. The binding sites of transcription factors and some elements are shown above the line. Hormone response elements binding sites are shown below the line. For a detailed description please refer to Laping et al. [14]. ThRE: thyroid hormone response factor element; ERE: estrogen response element; GRE: glucocorticoid response element, NF1: nuclear factor 1; AP2: activator protein 2; TIE: TGF-*β* inhibitory element; CRE: cAMP response element; NF*κ*B: nuclear factor *κ*B. Elements and features are not depicted to scale.

**Figure 4:**
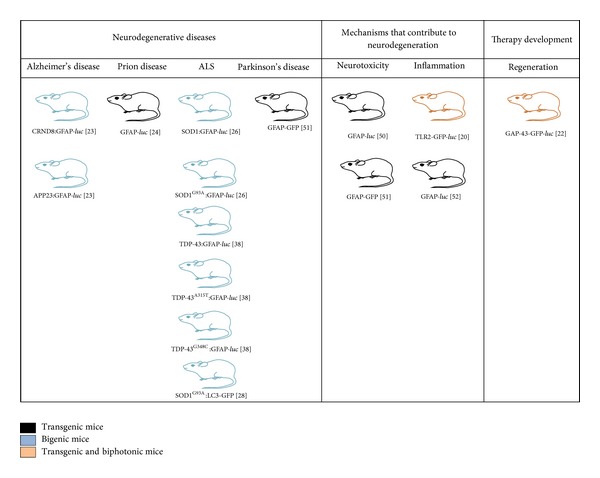
Bigenic and transgenic reporter mouse models that have been used for the study of neurodegeneration by* in vivo* fluorescent and bioluminescent optical imaging technology.

One promising approach by which* in vivo* optical imaging is contributing to the study of neurodegenerative diseases is through the use of transgenic mice in which a reporter gene (i.e., green fluorescent protein (GFP) or the enzyme* luciferase*) is under the control of an “activatable” promoter that acts as a disease biomarker. To this end, the glial fibrillary acidic protein (GFAP) promoter has been harnessed most often (Figure 3). GFAP is a major intermediate filament protein of astrocytes whose expression is highly regulated and is induced during astrocyte activation in response to multiple factors, notably from brain injury and disease including degenerative conditions [15–17]. The regulation of GFAP is most likely due to multiple sites within the promoter region of the gene. Although some promising sites have been identified, their significance and contribution to the overall regulatory control is still under investigation. Nevertheless, there are a plethora of sites for hormones, growth factors, inflammatory cytokines, and transcription factors (Figure 3). Additionally, epigenetic mechanisms such as phosphorylation and methylation are also likely to exert significant influence over GFAP transcription. Moreover, GFAP has also been shown to fluctuate under the circadian light-dark cycle [18]. In addition to GFAP, other similarly utilized promoters include heme oxygenase-1 promoter (HO-1), a marker for oxidative stress [19]; toll-like receptor 2 (TLR2) promoter, involved in the regulation of the inflammatory response of microglial cells [20]; microtubule associated protein 1 light chain 3 (LC3) promoter, a marker for autophagy [21]; and the growth-associated protein-43 (GAP-43) promoter, strongly upregulated in adult injured neurons as a part of the regenerative process [22] (Figure 4).

## 7. Alzheimer's Disease

Alzheimer's disease (AD) is the most common cause of dementia in adults and is characterized by the extracellular accumulation of amyloid plaques composed of aggregated amyloid *β* (A*β*) peptide, as well as intracellular neurofibrillary tangles composed of hyperphosphorylated and aggregated Tau protein. This, in turn, is highly neurotoxic. Research into the neuropathology of AD has been aided tremendously by generation of transgenic mice that accurately recapitulate the deposition of A*β*, often by overexpressing A*β* containing specific familial mutations in the amyloid precursor protein (APP) gene. Nevertheless, these models are also hindered by the fact that they do not show any overt clinical neurological symptoms of the disease and do not succumb to the deposition of A*β* in the brain. Therefore,* in vivo* diagnosis of AD pathology in the brains of these mice has proved to be challenging and most often can only be accomplished postmortem or through laborious learning and memory tests that are not only challenging but also quite often subjective. To delineate whether* in vivo* optical imaging would be a successful application for the study of AD, two widely used transgenic mouse models, transgenic lines APP23 and CRND8, were crossbred with reporter mice that express* luciferase* under the GFAP promoter to generate bigenic mice whose* luciferase* expression can be visualized [23]. In these bigenic mice, age and transgene dependent increases in* luciferase* signal were readily observed which correlated with the onset of robust A*β* deposition in the brain. In general, the CRND8:GFAP-*luciferase* mice showed a much earlier inflection in the bioluminescence emitting from the GAFP promoter than the APP23:GFAP-*luciferase* mice. Nevertheless, the signal emitted from these bigenic mice was far above the signal emitted from the control mice that were only GFAP-*luciferase*.* In vivo* optical imaging, therefore, permitted the diagnosis of a neurological disease in these mice in the absence of any overt signs of neurological dysfunction. Additionally, utilizing* in vivo *optical imaging technology, the visualization of accelerated deposition of A*β* in live APP23:GFAP-*luciferase* mice upon inoculation with brain homogenate derived from aged APP23 mice was possible [23]. Conceivably, the bioluminescence paradigm utilized in the study could be adapted to the study of any AD transgenic mouse lines to draw general conclusion about the molecular mechanisms contributing to the disease and permitting the early diagnosis of the disease in experimental animal models.

## 8. Prion Diseases

Prion diseases are rare, fatal neurodegenerative diseases caused by the misfolding and the subsequent replication of the infectious PrP^Sc^ molecule. The molecular mechanism(s) involved in the conversion of the cellular prion protein (PrP^c^) to the pathological isomer (PrP^Sc^), and the subsequent cascade of molecular events that contribute to the neurodegenerative process, remain elusive. Unlike other neurodegenerative diseases, wild-type mice can be inoculated with an infectious dose of prion inoculum and the course of disease progression can be monitored. Disease progression is highly reproducible when inoculating with a mouse-adapted prion strain and unlike many other neurodegenerative disorders, it can recapitulate neurological symptoms along with the pathology that is characteristic of the disease in humans. For this reason, prion models are potentially very useful for evaluating biomarkers of neuronal health and testing neuroprotective therapeutics. Astrocytic gliosis occurs simultaneously with prion replication thus permitting the use of transgenic GFAP-*luciferase* to monitor the progression of prion disease. To date, the application of* in vivo* optical imaging technology to the study of prion diseases has shown that the Rocky Mountain Lab (RML) scrapie strain in mice can be diagnosed at ~55 days after intracranial inoculation, which represents half the time required for the emergence of clinical symptoms, thus providing an early diagnostic criterion [24]. Alternate routes of prion infection that involve prion neuroinvasion from peripheral tissues, such as intraperitoneal inoculation and oral gavage, also resulted in detectable bioluminescent signals. Moreover, an inverse relationship was observed between the dose of prion inoculum administered and the point of bioluminescence inflection that was observed, relative to mock treated mice, over a wide range of prion dilutions. This study shows that alterations to bioluminescence signals between infected and control transgenic mice can indeed serve as a semiquantitative surrogate biomarker of prion replication. Undoubtedly,* in vivo* optical imaging technologies provide a new window of opportunity to test therapeutic interventions and visualize their effect on the onset of disease or progression. Moreover, this also affords the opportunity to optimize and refine classical bioassays by requiring fewer mice and shorter experimental time-courses. It is tempting to speculate whether genetically engineered mice with higher levels of* luciferase* expression would provide greater sensitivity and be conducive for earlier detection of astrocytic gliosis in parallel with the earliest replication of prions following inoculation [24].

## 9. Amyotrophic Lateral Sclerosis

Amyotrophic lateral sclerosis (ALS) is an adult-onset neurological disorder characterized by the progressive degeneration of motor neurons leading to muscle weakness, atrophy, paralysis, and subsequently death. The lifespan of individuals diagnosed with the clinical onset of ALS is often just five years. The pathological events contributing to the loss of motor neurons and the exact pattern of ALS spread are not fully understood. Novel findings utilizing* in vivo* optical imaging and bigenic reporter mice that possess both GFAP-*luciferase* and the SOD1^G93A^ mutation have contributed new insights into the pathobiology of ALS. In general, experimental animals possessing the human SOD1 mutation G93A develop features that resemble human familial and sporadic ALS [25]. In bigenic mice (SOD1^G93A^:GFAP-*luciferase*),* in vivo* optical imaging revealed that there are several successive stages of repeated increases in the expression of GFAP [26]. The first round of GFAP-*luciferase* increase corresponded with the asymptomatic stage at 25–30 days with prominent signal emanating from the lumbar spinal cords projections and peripheral neurons (projection areas of sciatic nerves). The second round corresponded with the clinical onset of the disease (85–90 days) which is characterized by distinct behavioral deficits and hind-limb paralysis. The peak signal at 113 days corresponded precisely with the induction of hind-limb paralysis. In the second round, prominent GFAP-*luciferase* signals emanated once again from peripheral sciatic neurons and Schwann cells. The authors suspect that the first round of GFAP promoter activation was most likely due to the expression of GFAP in astrocytes and glial progenitor cells, whereas the second round of promoter activation was most likely due to the activation of astrocytes in response to the ensuing pathology. In general, these studies revealed that toxicity to motor neurons in ALS was not noncell autonomous and that populations of nonneuronal cells, perhaps glial cells, can also affect the viability of motor neurons.

*In vivo* optical imaging of the SOD1^G93A^:GFAP-*luciferase* mice also showed an increased signal contribution from the corticospinal tract and upper motor neurons near the end stages of the disease [26]. Further* ex vivo* imaging of the affected brains to delineate the specific region(s) of signal occurrence confirmed that the signals mainly arose from the cortex and brainstem areas. These particular regions are implicated in the control of respiratory functions and the swallowing reflex, which suggests that damage within this region may contribute to the dramatic weight loss and breathing difficulties that is often associated with ALS [26]. Imaging also provided some evidence for “dying-back” neuropathy, or denervation in ALS, which may be initiated by the loss of neuromuscular junctions [27]. Recapitulation of the SOD1^G93A^:GFAP-*luciferase* neuropathy with SOD1:GFAP-*luciferase* mice that have undergone precise mechanical denervation using the cut-and-crush method of sciatic nerve injury provided some credible evidence for this hypothesis [26].

Analogous to the cellular role played by the ubiquitin-proteasome pathway, autophagy is considered to prevent the accumulation of abnormal proteins that may be toxic to the cell. Nevertheless, in ALS pathology, autophagy could also be involved in the process of motor neuron death. Microtubule associated protein 1 light chain 3 (LC3) is a marker for autophagy and bigenic mice possessing the fusion of the promoter region of LC3 to GFP and also the G93A mutant of human SOD1 has been generated in order to monitor* in vivo* autophagy in a mouse model of ALS [28].* In vivo* optical imaging of SOD1^G93A^:LC3-GFP at presymptomatic (10 weeks), early symptomatic (17 weeks), and late symptomatic (19 weeks) stages of the disease revealed a strong fluorescent signal* in vivo* over the T_3_–S_1_ level at 17 and 19 weeks of age in the double transgenic mice.* Ex vivo* autophagy imaging of spinal cord sections also showed a progressive increase of the fluorescence signal from 17 to 19 weeks in these mice in the anterior horn at the *L*_4-5_ level, and the fluorescence signals were clearly observed in the gray matter of the spinal cord with a progressive increase of the signal and decreases in large motor neurons. Taken together, these results suggest that although the activation of autophagy may be induced during the onset of ALS, the fusion of the autophagosome to the lysosome may become insufficient at the end stages of the disease, possibly contributing to motor neuron cell death [28].

The occurrence of ALS and frontotemporal lobar degeneration with ubiquitin inclusions (FTLD-U) in some families, and the discovery that the transactive response DNA-binding protein 43 (TDP-43) is present in the cytoplasmic aggregates of both diseases, provided the first set of clues that the two diseases may share a common underlying mechanism [29]. TDP-43 is a DNA/RNA binding protein that contains an N-terminal domain, two RNA-recognition motifs, and a glycine-rich C-terminal domain thought to be important in the mediation of protein-protein interactions [30, 31]. It serves a plethora of cellular functions but its implication in neurodegenerative diseases was primarily substantiated by the discovery of dominantly inherited missense mutations in TDP-43, which are present in patients with familial form ALS [21, 32–36]. Additionally, in neurodegenerative diseases, TDP-43 can be found in cytoplasmic ubiquitinated inclusions, where it shows poor solubility, hyperphosphorylation, and cleavage into smaller fragments [29]. Early mouse models expressing wild-type TDP-43 or mutant TDP-43 (A315T and M337V) exhibited early paralysis followed by death [37]. Moreover, many of these transgenic animals also exhibited increased ubiquitination of TDP-43 without the accumulation in inclusion bodies. Altogether, these observations raised questions about the validity and the usage of these animals as appropriate experimental models for the study of human forms of ALS. Many of these characteristics were primarily attributed to the high-level of neuronal expression of the transgene. Therefore, to better recapitulate the human version of the disease, alternate rodent models have been generated that show not only ubiquitous expression of the transgene, but also, more importantly, moderate levels, mainly due to the transgenes being under the control of their own promoters [38].* In vivo* optical imaging of bigenic versions of these alternate rodents (i.e., TDP-43:GFAP-*luciferase*, TDP-43^A315T^:GFAP-*luciferase*, and TDP-43^G348C^:GFAP-*luciferase*) showed that astrocytes are activated as early as 20 weeks in the brain, during a 52-week examination period, in TDP-43^G348C^:GFAP-*luciferase *mice. Moreover, the induction of astrogliosis in the brain and the spinal cord of all three bigenic models preceded the appearance of cognitive and motor abnormalities by up to 6–8 weeks.

## 10. Neuronal Damage Arising from Trauma

One of the primary causes of CNS neuronal damage is trauma to the brain which can initiate chronic molecular events that may be important epigenetic factors that predispose an individual to neurodegenerative conditions such as Alzheimer's disease [39, 40], Parkinson's disease [41], and ALS [42, 43], at a later time in life [44–46]. Emerging evidence also suggests that mild traumatic brain injury (TBI), which consists of concussive and mild concussive trauma, such as those encountered during sporting activities, can provoke a distinctive neurodegenerative state known as chronic traumatic encephalopathy (CTE) [47–49]. Trauma to the brain consists of the primary injury that disrupts brain tissue, followed by a cascade of secondary events that may spread by multiple molecular mechanisms. Secondary injuries consist of molecular events such as blood-brain-barrier (BBB) disruption, edema, oxidative stress, excitotoxicity, inflammation, and cell death. Clinical presentation of secondary injuries is usually delayed and, therefore, can be sensitive to therapeutic intervention. As such, secondary injury processes may serve as viable option(s) for imaging and therapeutic targets for the diagnosis and treatment of CNS neuronal damage caused by trauma. Some of the specific mechanisms of CNS neuronal injury that have been examined using* in vivo* optical imaging technology include neuronal excitotoxicity and inflammation. Insights gained from these studies can contribute to a better understanding of the molecular mechanisms associated with the secondary injury caused by trauma to the brain and, also, how best to curb these pathological features in an effort to circumvent the probability of developing a neurodegenerative pathology at a later time.

Trauma to the CNS may lead to excitotoxic events in the brain. Excitotoxicity is defined as cell death resulting from the toxic actions of excitatory amino acids (EAA). Since glutamate is the major excitatory neurotransmitter in the mammalian CNS, neuronal excitotoxicity usually refers to the injury and/or death of neurons arising from prolonged exposure to glutamate and the associated excessive influx of ions into the cell through glutamate-mediated receptors. The resulting ion overload (i.e., Ca^2+^) is particularly neurotoxic, leading to the activation of enzymes that degrade proteins, membranes, and nucleic acids. The overactivation of glutamate receptors can also impair cellular ion homeostasis, activate nitric oxide synthesis, generate free radicals, and induce programmed cell death. In experimental animal models, excitotoxicity can be induced through treatment with kainic acid (KA), a potent agonist for a subtype of glutamate receptors.* In vivo* optical imaging of excitotoxicity has been delineated through both GFAP-GFP and GFAP-*luciferase* mice. In these mice, significant elevation of GFAP signal was detected in the brain after subcutaneous treatment with KA [50, 51]. Additionally, in the GFAP-GFP mice, symptoms of Parkinson's disease were induced by the neurotoxicant 1-methyl-4-phenyl-1,2,3,6-tetrahydropyridine (MPTP). The effect of MPTP was also visualized after subcutaneous injection of the agent [51]. These reporter mouse models can therefore serve as useful tools to study the neuropathological consequences of excitotoxicity and neurotoxicants. Specifically, these models may permit the identification of key upstream molecular events that instigate or contribute to neuronal damage, which in turn will provide not only novel insights into the molecular basis of how neuronal cells die but also potential approaches for therapeutic intervention by uniquely targeting mechanisms involved in excitotoxic/neurotoxic signaling cascade.

Paradoxically, the inflammatory response can either aggravate or ameliorate the ensuing neuropathology associated with trauma to the brain. However, since the inflammatory response parallels that of secondary tissue injury, much interest has focused on the possibility of minimizing, or altogether arresting, certain components of inflammation in order to reduce secondary damage. Research from such a venture has broad applicability and is pertinent to the study of various neurodegenerative diseases. Several CNS resident cells, such as astrocytes and microglia, have innate inflammatory capacity and the live imaging of the activation of these cells has contributed some novel findings to the inflammatory mechanism operating under neurodegenerative conditions. The live imaging of inflammation in bigenic reporter mice (GFAP-*luciferase*) revealed that sex and estrogen levels are strong determinants of astrocyte activation/response caused by cerebral ischemia [52]. Following cerebral ischemia, GFAP-signals were markedly stronger in female transgenic mice than in males. However, these signals were diminished upon the entry of female mice into estrus or upon the pharmacological application of estrogen. Additional findings from this work suggest that the extent of the ischemia, based on the degree of signal intensity, is dictated by the size of the injury only in the male mice. No such correlation was observed in any of the experimental groups of female GFAP-*luciferase* mice used in the study.

The inflammatory response mediated by microglial cells can be regulated by Toll-like receptor 2 (TLR2) activation. Within the mouse brain, TLR2 expression is very low but is dramatically upregulated in response to infection and/or injury to the brain [53, 54]. Nevertheless, the mechanisms behind TLR2 activation, the long term consequence of activation, and brain region specific expression patterns of TLR2 are unknown. For these purposes, the TLR2-GFP-*luciferase* transgenic mice have provided some much needed understanding to the microglial activation process [20]. In a model of ischemia, the TLR2-GFP-*luciferase* mice showed TLR2 activation as early as 6 hours after the ischemic event. Interestingly, the activation was initially observed in the olfactory bulb (OB), even preceding its expression in the area of ischemic lesion. Moreover, longitudinal monitoring of TLR2 activation showed that the signal was detectable over the period of several months after the initial ischemic attack, implying that postischemic inflammatory process is much longer than previously understood. The biphasic nature of microglia activation (acute activation in OB followed by chronic activation at the site of ischemic lesion) was suggested to be a result of the distinct neuroanatomical location maintained by the OB. Specifically, the OB is located at a region that is considered to be at the interphase between the external environment and the brain. Perhaps this distinct location permits the expression of a unique subclass of microglia that may exist in a perpetually primed or alert state. This hypothesis was further supported by the parallel activation of TLR2 signal in the OB and at the site of intracranial inoculation of LPS. Furthermore, the OB was also able to translate TLR2 response and microglial activation signals, caused by inhalation of LPS, from the external environment into the brain.

## 11. *In Vivo* Optical Imaging for Diagnostic and Therapeutic Purposes

Drug discovery and evaluating the effectiveness of newly developed therapies are a priority that can be promptly addressed through the use of* in vivo* optical imaging. For example, novel protective monoclonal antibodies were discovered in mice infected with a bioluminescently tagged influenza A virus through binding of the hemagglutinin H1 and H5 subtypes [55]. Additionally, to assess drug efficacy, tumour response to Gefitinib, an epidermal growth factor receptor (EGFR) tyrosine kinase inhibitor, was evaluated in mice injected with fluorescently labeled tumourigenic A549 cells and found to reduce tumour size over time [56]. Similarly, a mouse model with stainless steel implants in the knee was inoculated with methicillin-sensitive* Staphylococcus aureus* (MSSA) to determine the optimal antibiotic use at different doses [57]. Therefore, it is reasonable to assume that the use of* in vivo* optical imaging and reporter mice technology for the study of neurodegenerative diseases will undoubtedly provide a reliable avenue for the development of novel diagnostic assays that show both improved sensitivity and specificity over current options. Moreover, these applications can also contribute towards the development of novel, disease-modifying therapies whose delivery and efficacy can be monitored in a longitudinal manner, permitting the use of less experimental animals and minimizing the variability that would emerge from using large sample sizes.

Nevertheless, the major hurdle for* in vivo* optical imaging, with respect to diagnostic and therapeutic molecule development for neurodegenerative diseases and disorders, remains the delivery of molecular agents across the restrictive BBB. The BBB ensures restrictive passage of molecules to the CNS in order to maintain proper functioning environment for the brain. Free passage of molecules would, therefore, disrupt intricate brain homeostasis. Passage of potential molecules across the BBB is also further hindered by the addition of fluorescent moieties or contrast agents that would be required for direct visualization. Not surprisingly, many of the molecules that have been developed are used to target receptors on the endovascular region which are upregulated during many pathological events of the brain. Nevertheless, one particular ligand that has been successfully evaluated, particularly using* in vivo* optical imaging technology, as a diagnostic tool for AD is the oxazine dye AOI987 [58, 59]. AOI987 has a low molecular weight, readily traverses the BBB, and shows high affinity towards A*β* plaque. It is well demonstrated that A*β* deposition precedes and most likely is involved in the induction of neuronal atrophy. Therefore, the deposition and subsequent quantification of A*β* load in the brain of affected individuals are imperative as detection of amyloid deposition may be the first step(s) towards diagnosis and subsequent optimization of treatment strategies for the ensuing neuropathology. Apart from the aforementioned properties, AOI987 absorbs and emits in the near infrared (NIR) fluorescence spectrum thus minimizing the impact of tissue autofluorescence and light-scattering that would be otherwise observed from dyes with a shorter absorption and emission spectrum. Another ligand that has been studied using* in vivo* optical imaging is the curcumin-derived NIR fluorescent probe CRANAD-2 which also shows a high affinity for A*β* [60]. Uniquely, upon intercalation with amyloid plaques, the probe not only increases in fluorescence and quantum yield but also undergoes a shift in the emission spectra by 90 nm. This particular spectral feature of CRANAD-2 is particularly intriguing as it may offer the ability to discriminate amyloid-bound probe from unbound probe, thereby enhancing the target-to-background signal. The aberrant aggregation of proteins/peptides is a common theme among most age related neurodegenerative diseases, including Parkinson's disease, Huntington's disease, ALS, and FTLD. Although the specific protein aggregates and the downstream cellular factors that are vulnerable differ, shared disease mechanisms are increasingly apparent among these diseases. It is, therefore, tempting to speculate whether the aforementioned ligands, their unique properties, and/or the technology used in their synthesis would be applicable for the detection and study of other CNS protein aggregation diseases using* in vivo* optical imaging technology. Versatile amyloid-specific fluorescent probes can have a very positive impact in the drug delivery and diagnostics fields for a wide range of neurodegenerative conditions and their delivery, function, and efficacy will undoubtedly be aided by* in vivo* optical imaging capabilities. Several other recent advances have been made that readily permit and/or assist in the transfer of molecules across the BBB, including potent viral vectors and nanoparticle technology, and it is foreseeable that these could be harnessed for* in vivo* optical imaging applications.

Apart from providing insights into the disease process reporter mice harboring transgenes can also provide highly specific mechanistic information on the biological specificity and efficacy of therapeutic agents. The most commonly used GFAP-*luciferase* transgene can provide novel insight into the degree of CNS injury recovery (or lack thereof) in response to a therapeutic. Another pertinent addition to the diversity of reporter mice currently available for* in vivo* optical imaging (Figure 4) is the GAP-43:*luciferase*-GFP mouse [22]. A unique property of this reporter mouse is that GAP-43 promoter is neuron specific and can, therefore, be utilized to sense neuronal response(s) to CNS injury. GAP-43 is a neuron specific phosphoprotein that is involved in neurite outgrowth and plasticity [61]. The induction of GAP-43 coincides with early neuronal development and is often considered to be mostly silent in the adult CNS. Nevertheless, it is strongly upregulated in the adult injured neuron and deregulation of the protein has also been observed in several neurodegenerative diseases [62–67]. Taken together, the upregulation of GAP-43 may represent a biomarker of regeneration within the adult CNS whose expression is induced in response to neuronal injury. Thus, the GAP-43:*luciferase*-GFP mouse may represent not only a suitable* in vivo* model to assess the innate regenerative process of the mature CNS but also a qualitative marker of the efficacy of therapeutic agents to promote this repair.

The therapeutic use of stem cells for regenerative and/or neuroprotective purposes has benefitted enormously by the application of* in vivo* optical imaging, specifically in tracking their survival. In one instance, neural stem cells (NSC) genetically engineered to overexpress glial-cell derived neurotrophic factor (GDNF) and also to express the* luciferase* gene have been tracked, quantified, and characterized* in vivo* upon grafting to the mouse brain in a Huntington's disease model (HD) [68]. Using* in vivo* optical imaging of* luciferase* gene expression, grafted GDNF-*luciferse *NSCs were shown to not only survive after the transplantation process but also migrate via the rostral migratory stream, the natural pathway for NSCs of the subventricular zone. The overexpression of GDNF by the NSCs, in turn, was shown to protect striatal projection neurons from an excitotoxic model of HD and to minimize the behavioral abnormalities associated with the disease [68].

## 12. Conclusion

The continuous refinement of currently available reporter gene mouse models for various neurodegenerative diseases, together with technical improvements in small animal* in vivo* optical imaging technology, has led to rapid progress in monitoring neurodegenerative disease pathobiology noninvasively in living animals. Insights from the pathophysiological processes related to disease initiation and progression can result in the identification of new molecular targets or treatment strategies. Novel functional imaging probes or contrasting agents directed towards disease-specific alterations have also been developed for improved diagnosis. Some of the most significant developments for* in vivo* optical imaging include the use and refinement of the bacterial* lux* gene cassette to eliminate luciferin use and the shift of fluorescent reporter emission wavelengths to the near-infrared spectrum to avoid tissue autofluorescence issues. Activatable probes that remain optically inactive until they are enzymatically cleaved by specific enzymes that are activated during disease are also revolutionizing our understanding of disease processes within living animals [1].

Although* in vivo* optical imaging is able to provide information on specific reporter location, it is not without a number of practical hurdles. One of the most challenging hurdles is the limited spatial resolution in tissue due to absorption and the difficulty in ascribing depth to the reporter signal as images are commonly acquired as planar and two-dimensional (2D). To circumvent this, other medical imaging modalities, such as microCT, can be used in tandem with optical imaging to generate three-dimensional (3D) images. Instrumentation and computer software are currently available to reconstruct images from a second modality (CT, MRI, or PET) with the 2D optical scans, using intricate algorithms based on the shape of the animal, how light passes through various thicknesses of tissue, and the scattering pattern, to precisely pinpoint where the signal of interest is originating from. Another technical advancement that has intriguing applications for* in vivo* optical imaging is improvements to the speed at which CCD cameras can process images that enables freely moving animals to be imaged. The In Actio Module, provided by Biospace Lab, records video through two CCD cameras at 45 frames per second; one is dedicated to imaging the subject, and the other records the light emitting reporter location and intensity. While still requiring much optimization, this technology opens the door to more exciting developments in free moving,* in vivo* animal imaging.

**Figure 5:**
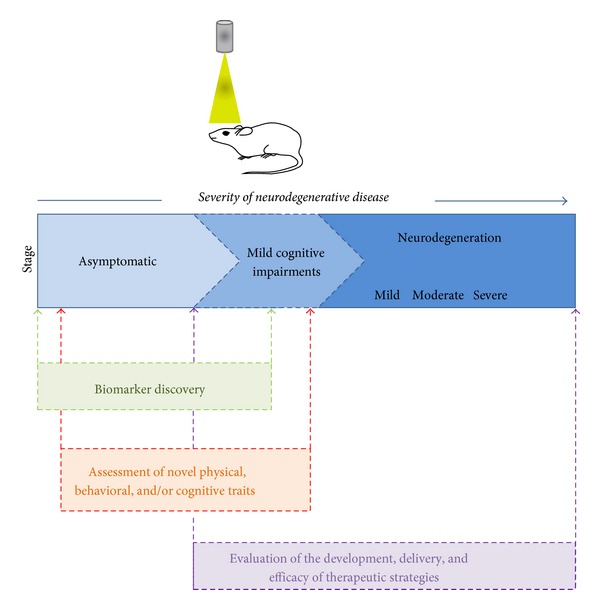
The selective advantage of utilizing* in vivo* optical imaging and small transgenic reporter animals in the study of neurodegeneration for the discovery of biomarkers and novel traits (physical, behavioral, and cognitive) and for visualizing the delivery and efficacy of therapeutic agents and strategies.

The ability of* in vivo* optical imaging technology to assess neurological disease states is gaining tremendous traction. The burgeoning choice of probes and animal models now require careful validation to confirm the specificity of imaging readouts. Undoubtedly, however, the establishment of effective biomarkers and end-points, capable of defining critical parameters such as genetic, metabolic, or behavioral signatures of specific neurodegenerative disorders, will provide faster, more effective, and less expensive ways to diagnose disorders (Figure 5). Moreover, this can also lead to better evaluation of drug efficacy and to the identification of potential subgroups of patients who are more likely to elicit enhanced responses from therapeutic intervention. Furthermore, the currently applied imaging protocols for disease diagnosis and therapy guidance need to be relentlessly replicated and subsequently standardized in order to compare and delineate experimental results between various research groups in order to draw definite conclusions.

*In vivo* optical imaging holds great promise not only in animal models but also for clinical imaging of the human brain. Advantages include the avoidance of radiation and radio-labeled tracers/agents in detection, the relative ease by which it can be performed without the need for complex surgical techniques, minor discomfort to the patient, and the relatively low cost for clinical/bedside implementation. To achieve success, major efforts in probe development and instrumentation is still required to overcome several technical challenges such as the potential toxicity of imaging probes or contrast agents given the larger quantities that must be administered to human patients, the ability to discriminate true cerebral signal from extracerebral contamination, and the degree of tissue penetrance. In the case of the latter, the dimensions of the imaging object (i.e., the human brain) also require powerful and large excitation source and an extremely sensitive detection camera. Additionally, the camera integration time must be optimized to sufficiently sample changes in fluorescence over time and thus measure fluorescence dynamics, which is imperative for longitudinal studies. A considerable challenge in the efforts to translate* in vivo* optical imaging findings from laboratory animals (i.e., rodents) to humans will be the need to perform similar set(s) of studies in nonhuman primates (NHP). NHP models offer sizeable advantages over those that use rodents and other small species because of their neurobiological similarity to humans and their longer life span, which makes it possible to study individual subjects over several years, an imperative requirement for neurological diseases. However, at the present time, this field is virtually unexplored. Ultimately, application of* in vivo* optical imaging of neurodegenerative diseases has tremendous potential to provide improved patient care and lead to the development of personalized precision medicine with greater efficacies and potentially fewer side effects.

## Figures and Tables

**Table 1:** Commonly used luciferase reporter systems. Information on some of the available luciferases for use in *in vivo *optical imaging experiments, including sources, emission wavelengths, substrates, and selective advantages for each.

Source	Emission wavelength (nm)	Substrate	Advantages
Bacteria (*Vibrio *and* Photobacterium *species)	478–545 (dependent on species)	FMNH2 + O_2_ + long chain fatty aldehyde	Exogenous substrate not required
Firefly (*Photinus pyralis*)	560	D-luciferin + ATP + O_2_	Most commonly used and modified for red-shifted emission
Sea pansy (*Renilla reniformis*)	480	Coelenterazine + O_2_	Different substrate allows multiplexing with firefly luciferase
Copepods (*Gaussia princeps *and others)	470	Coelenterazine + O_2_	Small size and secreted
Deep-sea shrimp (*Oplophorus gracilirostris*)	460	Furimazine + O_2_	Small size and secreted

## References

[1] Ntziachristos V (2010). Going deeper than microscopy: the optical imaging frontier in biology. *Nature Methods*.

[2] Hofstetter C, Flondor M, Boost KA (2005). A brief exposure to isoflurane (50 s) significantly impacts on plasma cytokine levels in endotoxemic rats. *International Immunopharmacology*.

[3] Choy G, O'Connor S, Diehn FE (2003). Comparison of noninvasive fluorescent and bioluminescent small animal optical imaging. *BioTechniques*.

[4] Taroni P, Pifferi A, Torricelli A, Comelli D, Cubeddu R (2003). In vivo absorption and scattering spectroscopy of biological tissues. *Photochemical and Photobiological Sciences*.

[5] Lichtman JW, Conchello JA (2005). Fluorescence microscopy. *Nature Methods*.

[6] Monici M (2005). Cell and tissue autofluorescence research and diagnostic applications. *Biotechnology Annual Review*.

[7] McNally JB, Kirkpatrick ND, Hariri LP (2006). Task-based imaging of colon cancer in the ApcMin/+ mouse model. *Applied Optics*.

[8] Mansfield JR, Gossage KW, Hoyt CC, Levenson RM (2005). Autofluorescence removal, multiplexing, and automated analysis methods for in-vivo fluorescence imaging. *Journal of Biomedical Optics*.

[9] Lichten CA, White R, Clark IB, Swain PS (2014). Unmixing of fluorescence spectra to resolve quantitative time-series measurements of gene expression in plate readers. *BMC Biotechnology*.

[10] Bentolila LA, Ebenstein Y, Weiss S (2009). Quantum dots for in vivo small-animal imaging. *Journal of Nuclear Medicine*.

[11] Hosseinkhani S (2011). Molecular enigma of multicolor bioluminescence of firefly luciferase. *Cellular and Molecular Life Sciences*.

[12] Aswendt M, Adamczak J, Couillard-Despres S, Hoehn M (2013). Boosting bioluminescence neuroimaging: an optimized protocol for brain studies. *PLoS ONE*.

[13] Close DM, Patterson SS, Ripp S, Baek SJ, Sanseverino J, Sayler GS (2010). Autonomous bioluminescent expression of the bacterial luciferase gene cassette (lux) in a mammalian cell line. *PLoS ONE*.

[14] Laping NJ, Teter B, Nichols NR, Rozovsky I, Finch CE (1994). Glial fibrillary acidic protein: regulation by hormones, cytokines, and growth factors. *Brain Pathology*.

[15] Eng LF, Ghirnikar RS, Lee YL (2000). Glial fibrillary acidic protein: GFAP-thirty-one years (1969–2000). *Neurochemical Research*.

[16] Middeldorp J, Hol EM (2011). GFAP in health and disease. *Progress in Neurobiology*.

[17] Martin A, Hofmann HD, Kirsch M (2003). Glial reactivity in ciliary neurotrophic factor-deficient mice after optic nerve lesion. *The Journal of Neuroscience*.

[18] Hajós F (2008). Changes in glial fibrillary acidic protein (GFAP) immonureactivity reflect neuronal states. *Neurochemical Research*.

[19] Marques CP, Cheeran MC, Palmquist JM, Hu S, Lokensgard JR (2008). Microglia are the major cellular source of inducible nitric oxide synthase during experimental herpes encephalitis. *Journal of NeuroVirology*.

[20] Lalancette-Hbert M, Phaneuf D, Soucy G, Weng YC, Kriz J (2009). Live imaging of toll-like receptor 2 response in cerebral ischaemia reveals a role of olfactory bulb microglia as modulators of inflammation. *Brain*.

[21] Gitcho MA, Baloh RH, Chakraverty S (2008). TDP-43 A315T mutation in familial motor neuron disease. *Annals of Neurology*.

[22] Gravel M, Weng Y, Kriz J (2011). Model system for live imaging of neuronal responses to injury and repair. *Molecular Imaging*.

[23] Watts JC, Giles K, Grillo SK, Lemus A, DeArmond SJ, Prusiner SB (2011). Bioluminescence imaging of A*β* deposition in bigenic mouse models of alzheimer's disease. *Proceedings of the National Academy of Sciences of the United States of America*.

[24] Tamgüney G, Francis KP, Giles K, Lemus A, DeArmond SJ, Prusiner SB (2009). Measuring prions by bioluminescence imaging. *Proceedings of the National Academy of Sciences of the United States of America*.

[25] Gurney ME, Pu H, Chiu AY (1994). Motor neuron degeneration in mice that express a human Cu,Zn superoxide dismutase mutation. *Science*.

[26] Keller AF, Gravel M, Kriz J (2009). Live imaging of amyotrophic lateral sclerosis pathogenesis: disease onset is characterized by marked induction of GFAP in schwann cells. *GLIA*.

[27] Fischer LR, Culver DG, Tennant P (2004). Amyotrophic lateral sclerosis is a distal axonopathy: evidence in mice and man. *Experimental Neurology*.

[28] Tian F, Morimoto N, Liu W (2011). In vivo optical imaging of motor neuron autophagy in a mouse model of amyotrophic lateral sclerosis. *Autophagy*.

[29] Neumann M, Sampathu DM, Kwong LK (2006). Ubiquitinated TDP-43 in frontotemporal lobar degeneration and amyotrophic lateral sclerosis. *Science*.

[30] Forman MS, Trojanowski JQ, Lee VM (2007). TDP-43: a novel neurodegenerative proteinopathy. *Current Opinion in Neurobiology*.

[31] Lagier-Tourenne C, Cleveland DW (2009). Rethinking ALS: the FUS about TDP-43. *Cell*.

[32] Kabashi E, Valdmanis PN, Dion P (2008). TARDBP mutations in individuals with sporadic and familial amyotrophic lateral sclerosis. *Nature Genetics*.

[33] Rutherford NJ, Zhang Y, Baker M (2008). Novel mutations in TARDBP(TDP-43) in patients with familial amyotrophic lateral sclerosis. *PLoS Genetics*.

[34] Sreedharan J, Blair IP, Tripathi VB (2008). TDP-43 mutations in familial and sporadic amyotrophic lateral sclerosis. *Science*.

[35] van Deerlin VM, Leverenz JB, Bekris LM (2008). *TARDBP* mutations in amyotrophic lateral sclerosis with TDP-43 neuropathology: a genetic and histopathological analysis. *The Lancet Neurology*.

[36] Yokoseki A, Shiga A, Tan C (2008). TDP-43 mutation in familial amyotrophic lateral sclerosis. *Annals of Neurology*.

[37] Wegorzewska I, Bell S, Cairns NJ, Miller TM, Baloh RH (2009). TDP-43 mutant transgenic mice develop features of ALS and frontotemporal lobar degeneration. *Proceedings of the National Academy of Sciences of the United States of America*.

[38] Swarup V, Phaneuf D, Bareil C (2011). Pathological hallmarks of amyotrophic lateral sclerosis/frontotemporal lobar degeneration in transgenic mice produced with TDP-43 genomic fragments. *Brain*.

[39] Magnoni S, Brody DL (2010). New perspectives on amyloid-*β* dynamics after acute brain injury: moving between experimental approaches and studies in the human brain. *Archives of Neurology*.

[40] Sharp DJ, Scott G, Leech R (2014). Network dysfunction after traumatic brain injury. *Nature Reviews Neurology*.

[41] Goldman SM, Tanner CM, Oakes D, Bhudhikanok GS, Gupta A, Langston JW (2006). Head injury and Parkinson's disease risk in twins. *Annals of Neurology*.

[42] Chen H, Richard M, Sandler DP, Umbach DM, Kamel F (2007). Head injury and amyotrophic lateral sclerosis. *The American Journal of Epidemiology*.

[43] Schmidt S, Kwee LC, Allen KD, Oddone EZ (2010). Association of ALS with head injury, cigarette smoking and APOE genotypes. *Journal of the Neurological Sciences*.

[44] Masel BE, DeWitt DS (2010). Traumatic brain injury: a disease process, not an event. *Journal of Neurotrauma*.

[45] Blennow K, Hardy J, Zetterberg H (2012). The neuropathology and neurobiology of traumatic brain injury. *Neuron*.

[46] Smith DH, Johnson VE, Stewart W (2013). Chronic neuropathologies of single and repetitive TBI: substrates of dementia?. *Nature Reviews Neurology*.

[47] McKee AC, Cantu RC, Nowinski CJ (2009). Chronic traumatic encephalopathy in athletes: progressive tauopathy after repetitive head injury. *Journal of Neuropathology and Experimental Neurology*.

[48] Dekosky ST, Blennow K, Ikonomovic MD, Gandy S (2013). Acute and chronic traumatic encephalopathies: pathogenesis and biomarkers. *Nature Reviews Neurology*.

[49] Jordan BD (2013). The clinical spectrum of sport-related traumatic brain injury. *Nature Reviews Neurology*.

[50] Zhu L, Ramboz S, Hewitt D, Boring L, Grass DS, Purchio AF (2004). Non-invasive imaging of GFAP expression after neuronal damage in mice. *Neuroscience Letters*.

[51] Ho G, Zhang C, Zhuo L (2007). Non-invasive fluorescent imaging of gliosis in transgenic mice for profiling developmental neurotoxicity. *Toxicology and Applied Pharmacology*.

[52] Cordeau P, Lalancette-Hébert M, Weng YC, Kriz J (2008). Live imaging of neuroinflammation reveals sex and estrogen effects on astrocyte response to ischemic injury. *Stroke*.

[53] Nguyen MD, Julien J, Rivest S (2002). Innate immunity: the Missing link in neuroprotection and neurodegeneration?. *Nature Reviews Neuroscience*.

[54] Laflamme N, Echchannaoui H, Landmann R, Rivest S (2003). Cooperation between toll-like receptor 2 and 4 in the brain of mice challenged with cell wall components derived from gram-negative and gram-positive bacteria. *European Journal of Immunology*.

[55] Heaton NS, Leyva-Grado VH, Tan GS, Eggink D, Hai R, Palese P (2013). In vivo bioluminescent imaging of influenza a virus infection and characterization of novel cross-protective monoclonal antibodies. *Journal of Virology*.

[56] Liu Z, Sun X, Liu H (2014). Early assessment of tumor response to gefitinib treatment by noninvasive optical imaging of tumor vascular endothelial growth factor expression in animal models. *Journal of Nuclear Medicine*.

[57] Niska JA, Shahbazian JH, Ramos RI (2012). Daptomycin and tigecycline have broader effective dose ranges than vancomycin as prophylaxis against a *Staphylococcus aureus* surgical implant infection in mice. *Antimicrobial Agents and Chemotherapy*.

[58] Hintersteiner M, Enz A, Frey P (2005). *In vivo* detection of amyloid-*β* deposits by near-infrared imaging using an oxazine-derivative probe. *Nature Biotechnology*.

[59] Hyde D, de Kleine R, MacLaurin SA (2009). Hybrid FMT-CT imaging of amyloid-*β* plaques in a murine Alzheimer's disease model. *NeuroImage*.

[60] Chongzhao R, Xiaoyin X, Raymond SB (2009). Design, synthesis, and testing of difluoroboron-derivatized curcumins as near-infrared probes for in vivo detection of amyloid-*β* deposits. *Journal of the American Chemical Society*.

[61] Benowitz LI, Routtenberg A (1997). GAP-43: an intrinsic determinant of neuronal development and plasticity. *Trends in Neurosciences*.

[62] de la Monte SM, Ng S-C, Hsu DW (1995). Aberrant GAP-43 gene expression in Alzheimer’s disease. *The American Journal of Pathology*.

[63] Martzen MR, Nagy A, Coleman PD, Zwiers H (1993). Altered phosphorylation of growth-associated protein B50/GAP-43 in Alzheimer disease with high neurofibrillary tangle density. *Proceedings of the National Academy of Sciences of the United States of America*.

[64] Teunissen CE, Dijkstra CD, Jasperse B (2006). Growth-associated protein 43 in lesions and cerebrospinal fluid in multiple sclerosis. *Neuropathology and Applied Neurobiology*.

[65] Parhad IM, Oishi R, Clark AW (1992). GAP-43 gene expression is increased in anterior horn cells of amyotrophic lateral sclerosis. *Annals of Neurology*.

[66] Ikemoto A, Hirano A, Akiguchi I (1999). Increased expression of growth-associated protein 43 on the surface of the anterior horn cells in amyotrophic lateral sclerosis. *Acta Neuropathologica*.

[67] Perrin FE, Boisset G, Docquier M, Schaad O, Descombes P, Kato AC (2005). No widespread induction of cell death genes occurs in pure motoneurons in an amyotrophic lateral sclerosis mouse model. *Human Molecular Genetics*.

[68] Pineda JR, Rubio N, Akerud P (2007). Neuroprotection by GDNF-secreting stem cells in a Huntington's disease model: optical neuroimage tracking of brain-grafted cells. *Gene Therapy*.

